# Hard Surface Biocontrol in Hospitals Using Microbial-Based Cleaning Products

**DOI:** 10.1371/journal.pone.0108598

**Published:** 2014-09-26

**Authors:** Alberta Vandini, Robin Temmerman, Alessia Frabetti, Elisabetta Caselli, Paola Antonioli, Pier Giorgio Balboni, Daniela Platano, Alessio Branchini, Sante Mazzacane

**Affiliations:** 1 CIAS Laboratory, Centre for the Study of physical, chemical and microbiological Contamination of Highly Sterile Environments, Department of Architecture, University of Ferrara, Ferrara, Italy; 2 Laboratory of Microbial Ecology and Technology, Ghent University, Ghent, Belgium; 3 Chrisal R & D Department, Lommel, Belgium; 4 Department of Medical Sciences, Microbiology Section, University of Ferrara, Ferrara, Italy; 5 Department of Infection Prevention Control and Risk Management, Ferrara University Hospital, Ferrara, Italy; 6 Department of Biomedical and Neuromotor Sciences, University of Bologna, Bologna, Italy; 7 Department of Life Sciences and Biotechnology, University of Ferrara, Ferrara, Italy; Graz University of Technology (TU Graz), Austria

## Abstract

**Background:**

Healthcare-Associated Infections (HAIs) are one of the most frequent complications occurring in healthcare facilities. Contaminated environmental surfaces provide an important potential source for transmission of many healthcare-associated pathogens, thus indicating the need for new and sustainable strategies.

**Aim:**

This study aims to evaluate the effect of a novel cleaning procedure based on the mechanism of biocontrol, on the presence and survival of several microorganisms responsible for HAIs (i.e. coliforms, *Staphyloccus aureus*, *Clostridium difficile*, and *Candida albicans*) on hard surfaces in a hospital setting.

**Methods:**

The effect of microbial cleaning, containing spores of food grade *Bacillus subtilis*, *Bacillus pumilus* and *Bacillus megaterium*, in comparison with conventional cleaning protocols, was evaluated for 24 weeks in three independent hospitals (one in Belgium and two in Italy) and approximately 20000 microbial surface samples were collected.

**Results:**

Microbial cleaning, as part of the daily cleaning protocol, resulted in a reduction of HAI-related pathogens by 50 to 89%. This effect was achieved after 3–4 weeks and the reduction in the pathogen load was stable over time. Moreover, by using microbial or conventional cleaning alternatively, we found that this effect was directly related to the new procedure, as indicated by the raise in CFU/m^2^ when microbial cleaning was replaced by the conventional procedure. Although many questions remain regarding the actual mechanisms involved, this study demonstrates that microbial cleaning is a more effective and sustainable alternative to chemical cleaning and non-specific disinfection in healthcare facilities.

**Conclusions:**

This study indicates microbial cleaning as an effective strategy in continuously lowering the number of HAI-related microorganisms on surfaces. The first indications on the actual level of HAIs in the trial hospitals monitored on a continuous basis are very promising, and may pave the way for a novel and cost-effective strategy to counteract or (bio)control healthcare-associated pathogens.

## Introduction

Healthcare-Associated Infections (HAIs) are one of the most frequent complications occurring in healthcare facilities and represent a problematic concern regarding the safety and quality of healthcare worldwide [1], as also stated in a recent report by the World Health Organization estimating hospital-wide prevalence in high-income countries at 8% [2]. The European Center for Disease Control point prevalence study confirmed that healthcare-associated infections are a major public health problem in Europe with a prevalence of 5.7% (4.5–7.4%) which means 81.089 (64.624–105.895) patients with one HAI for each day in European acute care hospitals [3]. In particular, this European survey reported a similar estimation of nosocomial infections for Italy and Belgium, where the percentage of patients with HAIs has been calculated as 6.3% (5.4–7.4%) and 7.1% (6.1–8.3%), respectively [1]. Based on this study, the estimated total annual number of patients with an HAI in European acute care hospitals in 2011–2012 was 3.2 million, albeit with a wide confidence interval from 1.9 to 5.2 million patients. Similar incidences were measured in the United States [4]. Besides human suffering, also impressive economic costs are related to HAIs management. Indeed, as reported by the Centers for Disease Control and Prevention (CDC), it has been estimated that the overall annual direct medical costs for healthcare-associated infections in hospitals ranges from 35.7 to 45 billion dollars in the United States [5]. In addition, the management, prevention and monitoring of HAIs nowadays still represents a challenge for healthcare facilities [6], [7].

The microorganisms most frequently isolated from HAIs are, in decreasing order, *Escherichia coli* (15.9%), *Staphylococcus aureus* (12.3%), *Enterococcus spp.* (9.6%), *Pseudomonas aeruginosa* (8.9%) *Klebsiella spp.* (8.7%), coagulase-negative staphylococci (7.5%), *Candida spp.* (6.1%), *Clostridium difficile* (5.4%), *Enterobacter spp.* (4.2%), *Proteus spp.* (3.8%) and *Acinetobacter spp.* (3.6%) [3].

A very controversial and debated question is the qualitative and quantitative role of the environment in the patient contamination process, particularly the role of confinement and furnishing surfaces. It is well known that surfaces act as reservoirs for microorganisms and could contribute to the transmission of hospital pathogens, increasing the risk of cross-contamination through indirect contact with the patient [8]–[10]. To reduce such risks, sanitation procedures are applied to every surface that directly or indirectly may come in contact with people. Despite experimental evidence suggesting that a reasonable use of disinfectants is recommended, their routine use is still controversial [11], [12]. Nevertheless, a proper surface disinfection is recommended by all international guidelines as an important procedure for preventing infections [13]–[17], and considerable evidence exist concerning the benefits of hospital cleanliness towards reducing HAIs [18]. Indeed, failure to ensure proper cleaning and sterilization or disinfection may lead to patient-to-patient transmission of pathogens [19].

However, the widespread use of chemical disinfectants presents risks towards the environment and the safety of personnel. It is clear that microorganisms can adapt to a variety of environmental physical and chemical conditions, and it is therefore not surprising that resistance to extensively used antiseptics and disinfectants has been reported [20], [21]. For these reasons, the importance of cleaning procedures that are aimed to control the load of pathogenic bacteria indicates that a new and sustainable strategy is necessary.

A very promising approach, as suggested by Falagas & Makris in 2009, is the use of non-pathogenic microorganisms, namely probiotics and defined as living microorganisms able to confer a health benefit on the host, to colonize hard surfaces in order to counteract the proliferation of other bacterial species [22], according to the competitive exclusion principle (Gause's law) [23]–[25]. This concept has been designated as biocontrol when the application is antagonistic towards a certain pathogen [26], and has already successfully been applied to the abatement of Legionella in water systems [27].

Several investigators have pointed to evidence that probiotic type microorganisms and their biosurfactants may antagonize the growth of nosocomial pathogens on inanimate surfaces [28]–[32]. However, the actual application of probiotic type microorganisms on hard surfaces as a cleaning procedure has never been tested. Therefore, this study evaluates the effects of microbial cleaning of hard surfaces in hospitals on the presence and/or survival of HAI-related microorganisms on such treated surfaces. In order to facilitate the statistic meaningfulness of the results, the study was performed in three independent hospitals (one in Belgium and two in Italy). Comparison was made with chemical cleaning and disinfection protocols. This study aims to provide sufficient data to conclude whether the technique of biocontrol of hospital surfaces can act as a sustainable alternative to chemical disinfectants.

## Methods

### Preliminary tests

Prior to the actual field trials in the hospitals, a number of preliminary tests were done at Ghent University, Ferrara University and AZ Lokeren hospital in order to determine the most suitable formulation of the microbial cleaning products to be used. Mainly the identity and concentration of the micro-organisms used in the cleaning products were chosen in view of the average microbiological load on hard surfaces, pH, temperature and humidity. The microbial cleaning products used in the field trials comprised spores of *Bacillus subtilis*, *Bacillus pumilus* and *Bacillus megaterium*, with a fixed quantity of 5×10^7^ CFU per ml of product concentrate. All products were manufactured by Chrisal (Lommel, Belgium) and supplied to AZ Lokeren by Chrisal and the two Italian hospitals by Copma scrl. (Ferrara, Italy).

In order to prevent bias in the eventual hospital trials due to the detergents in the products used, several field trials in the AZ Lokeren hospital were performed to compare the effect between the microbial and non-microbial version of the products to be used in the hospital trials (data not shown).

### Ethics Statement

The study protocol was reviewed and approved by the local Ethics Committees. The trials in the two Hospitals residing in Ferrara (Sant'Anna and San Giorgio) were approved by the Ethics Committee, named Comitato Unico della Provincia di Ferrara (Unique Committee of the Ferrara Province), of the Azienda Ospedaliero-Universitaria of Ferrara (Ferrara, Italy). For the AZ Lokeren setting, the study was evaluated and approved by the AZ Lokeren Ethisch Comité (Lokeren, Belgium). The two Ethics Committees stated that a formal authorization was not necessary because the probiotic products would not be directly administered to patients but exploited for cleaning of hospital surfaces only. For this reason, the Committees waived the need for written informed consent from participants because of the observational nature of the study.

### Hospital trial setup

Three independent hospital trials, separated in time and location were performed. In each trial setting, comparison was made between cleaning with microbial cleaning products and the conventional hygiene protocols (using chemical cleaning products and disinfectants). Control cleaning products in AZ Lokeren consisted of chemical detergents (Ecolab, Groot-Bijgaarden, Belgium) and in both Italian hospitals chlorine based detergents (Actichlor for all washable surfaces, Diversey S.p.A., Italy) were applied. The microbial cleaning products in all three hospitals comprised a floor cleaner, interior cleaner and bathroom cleaner (Chrisal, Lommel, Belgium). Comparison between control and microbial cleaning was made both over time and on units with identical infrastructure within the hospital (e.g. two floors of geriatrics in AZ Lokeren). Except for the products, all other parameters related to the cleaning procedures (e.g. frequency, equipment) were the same between control and microbial cleaning. Cleaning in AZ Lokeren was performed according to the existing hygiene protocol of the hospital, and cleaning in the two Italian hospitals was performed according to the Probiotic Cleaning Hygiene System (PCHS) by Copma scrl. Cleaning staff was not aware whether or not they were operating with microbial cleaning products.

### Description of hospital wards

#### San Giorgio Rehabilitation Hospital

The San Giorgio hospital (total surface 12,300 m^2^) is a centre for the rehabilitation of acquired severe brain-damaged disorders. The wards involved in this study are distributed on a total of three floors, with each floor (4,100 m^2^) consisting of two identical and specular wards, namely the Severe Brain-damaged Unit and the Rehabilitation Medicine Unit. These two wards are separated by a central corridor and each consists of 22 recovery rooms (28 m^2^) with two beds and a toilet (4.2 m^2^). In these settings, treated surfaces consisted of room, corridor, and floors and toilets. In addition, cleaning procedures and samplings were also performed in the six gymnasiums used for patient rehabilitation.

#### Sant'Anna University Hospital

The wards involved in the study within the Sant'Anna University Hospital are referred to as an in-patients general medicine ward, consisting of two identical areas (named S- and T-Area, each with a surface of 550 m^2^), and an out-patient ward containing the Ophthalmology/Cardiology and Orthopedics departments (286 m^2^ each, with rooms of 22 m^2^). Recovery rooms (a total of 20, with a surface of 38 m^2^ each) and toilets (10 m^2^) were monitored during microbiological samplings.

#### AZ Lokeren Hospital

In the Lokeren hospital setting, two structurally identical (500 m^2^ per unit) geriatrics units were involved in the study. The wards host elderly people suffering from a wide range of illness and pathologic states, including surgery, daycare, and some cases in which the treatment of infections is needed.

### Microbiological tests

The effect of the microbial and conventional cleaning products on HAI related microorganisms on surfaces was assessed by means of surface sampling and culture-based microbiology tests. The following HAI-related microorganisms were monitored: *Staphylococcus aureus* and *Escherichia coli* in all three hospitals; *Clostridium spp*. in AZ Lokeren hospital; *Candida albicans* in Sant'Anna and San Giorgio hospital. During six weeks in Sant'Anna University hospital, 24 weeks in AZ Lokeren hospital and 66 weeks in San Giorgio Hospital, a total of 20000 microbiological samples were collected (between six and eight hours after cleaning) from a broad variety of surfaces such as floors, doors, showers, window sills, toilets, sinks, made of stone, wood, plastic, glass or metal.

All surface samples were performed in triplicate by contact RODAC (Replicate Organism Detection and Counting) plates, used for microbiological monitoring surface equivalent to 24 cm^2^ of the surface. The following growth media were used: McConkey Agar (BBL MacConkey Agar, BD) as selective and differential medium for the detection and enumeration of *Enterobacteriaceae* (especially the group of coliform bacteria); Baird Parker Agar (Merck Millipore Baird-Parker Agar) as moderately selective and differential medium for the detection and enumeration of coagulase-positive staphylococci, and used for detecting *Staphylococcus aureus*; Clostridium difficile agar (BBL Clostridium difficile Agar, BD) for the selective detection of *Clostridium* difficile; and Sabouraud Dextrose Contact Agar added with chloramphenicol (Merck Millipore) as selective medium for detection and enumeration of *Candida albicans*. Incubation was done aerobically at 37°C (48 h–72 h) for MacConkey, Baird Parker, Cetrimide Agar and Sabouraud Dextrose Contact Agar, and anaerobically using anaerobic jars (GasPak, BD) at 37°C (72 h) for *Clostridium difficile* agar. Colony Forming Units (CFU) on all agar plates were manually counted after their respective incubation period. Occasionally isolates from the above agar plates were identified to check the selectivity of the used media. Identification of isolates was assessed by API 20 E (bioMérieux, Inc, Durham, NC, USA), BBL Enterotube II (BD Diagnostic Systems) for *Escherichia coli*, API Staph (bioMérieux, Inc) for *Staphylococcus aureus*, and API 20 C Aux (bioMérieux, Inc) for *Candida albicans*.

### Antibiograms

The protocol used was based on the Kirby-Bauer disk diffusion antimicrobial susceptibility test [33]–[35], following the criteria outlined by the Clinical and Laboratory Standard Institute (CLSI) [36], [37]. Briefly, a 90 mm Mueller-Hinton agar plate was inoculated with a suspension of *Bacillus subtilis* (ATCC 6633), or *Bacillus* strains isolates, corresponding to a 0.5 McFarland standard. After 15 minutes, a paper disk (Oxoid Ltd., Thermo Scientific, Basingstoke, Hampshire, United Kingdom) impregnated with a known concentration of an antimicrobial compound was added, and plates were incubated at 37°C for 24–48 hours. The zones of inhibition (expressed in mm) were measured, and the interpretation of results was based on CLSI reference criteria (Table S1) [38].

### Molecular analyses

The presence of antimicrobial resistance genes in the *Bacillus* collected isolates was evaluated by a real time quantitative PCR (qPCR) method. Microbial genomic DNA was extracted from each isolate by the QIAmp UCP Pathogen Mini Kit (Qiagen, Hilden, Germany), adjusting the instruction of the manufacturer for optimal lysis of Gram-positive bacilli and spores. Two micrograms of extracted DNA were analyzed by the Microbial DNA qPCR Array for Antibiotic Resistance Genes (Qiagen, Hilden, Germany), using an Applied Biosystem 7300 instrument and following the manufacturer's indications. The analysis was performed on the strains originally present in the cleaning products and on 20 different isolates from the different hospital settings involved in the study. Microbial DNA-free water (Qiagen) and Microbial DNA Positive Control (Qiagen) were used as negative and positive controls, respectively. DNA from *E. coli* JM101 strain was also used as a negative control. Preliminary tests showed that the sensitivity of the assay was about 10 copies for each target gene.

### Statistics

The statistical analysis was based on the generalized linear models for repeated measures. This method combines the repeated measures of a marginal model with the generalized linear model for residuals following the Poisson distribution and the logarithm as link function. Comparisons between follow up and basal times were performed by using Wald Statistics with the Sidak correction for multiple comparisons. All statistical analyses were performed using the SPSS v 19.0 software (IBM Corp., Armonk, NY; USA).

## Results

Prior to hospital trials, a broad range of *in vitro* and small scale field trials were performed in order to determine both the best possible test protocols and product formulations for the actual study, as well as to provide relevant information to the ethics committees. The focus of the study was on microbiological counts and no evaluations of actual dirt removal were assessed. The use of *Bacillus* strains in the cleaning products showed a much higher microbiological influence on hard surfaces compared to other genera (such as *Lactobacillus* and *Pseudomonas*), mainly due to their sporulation capacity (data not shown). Based on the average total count values of several types of hard surfaces in hospitals, the optimal concentration of *Bacillus* spores to be employed in the product formulations was determined. Before starting the hospital trials, we decided that the amount of product bacteria to be left on surfaces upon cleaning should be of the same magnitude as the average total count observed before the microbial cleaning application. Finally, a number of laboratory scale tests demonstrated that the same product formulations, with or without the addition of the *Bacillus* spores, produced significantly different effects on pathogen load on the treated surfaces.

The study was conducted in three different hospitals, and approximately 20000 microbial surface samples were collected. The microbial analyses focused on *Staphylococcus aureus*, coliform bacteria, *Clostridium difficile*, and *Candida albicans* as representatives or indicators for proper hygiene and HAI-related microorganisms. In order to allow proper comparison among the different hospital settings, results are presented as relative values to the control, which is referred to the value of microbial surface contamination (CFU count) at the beginning of the trials (week 0), as shown in Tables S2, S3, S4, S5.

Notably, the effect of microbial cleaning on the different HAI-related pathogens displayed similar trends among the three hospitals under investigation. In the Sant'Anna University hospital, the microbial sampling lasted six weeks, whereas the other two hospital were monitored until week 24 (AZ Lokeren) and week 66 (San Giorgio Hospital). However, because no significant differences in counts were observed between week 24 and 66 at the San Giorgio Hospital, data presented here are limited to 24 weeks. In addition, during trials the total microbial count was also measured, but no significant changes were observed during the entire period spanning the hospital trials (data not shown).

### Coliforms and *E. coli*

**Figure 1:**
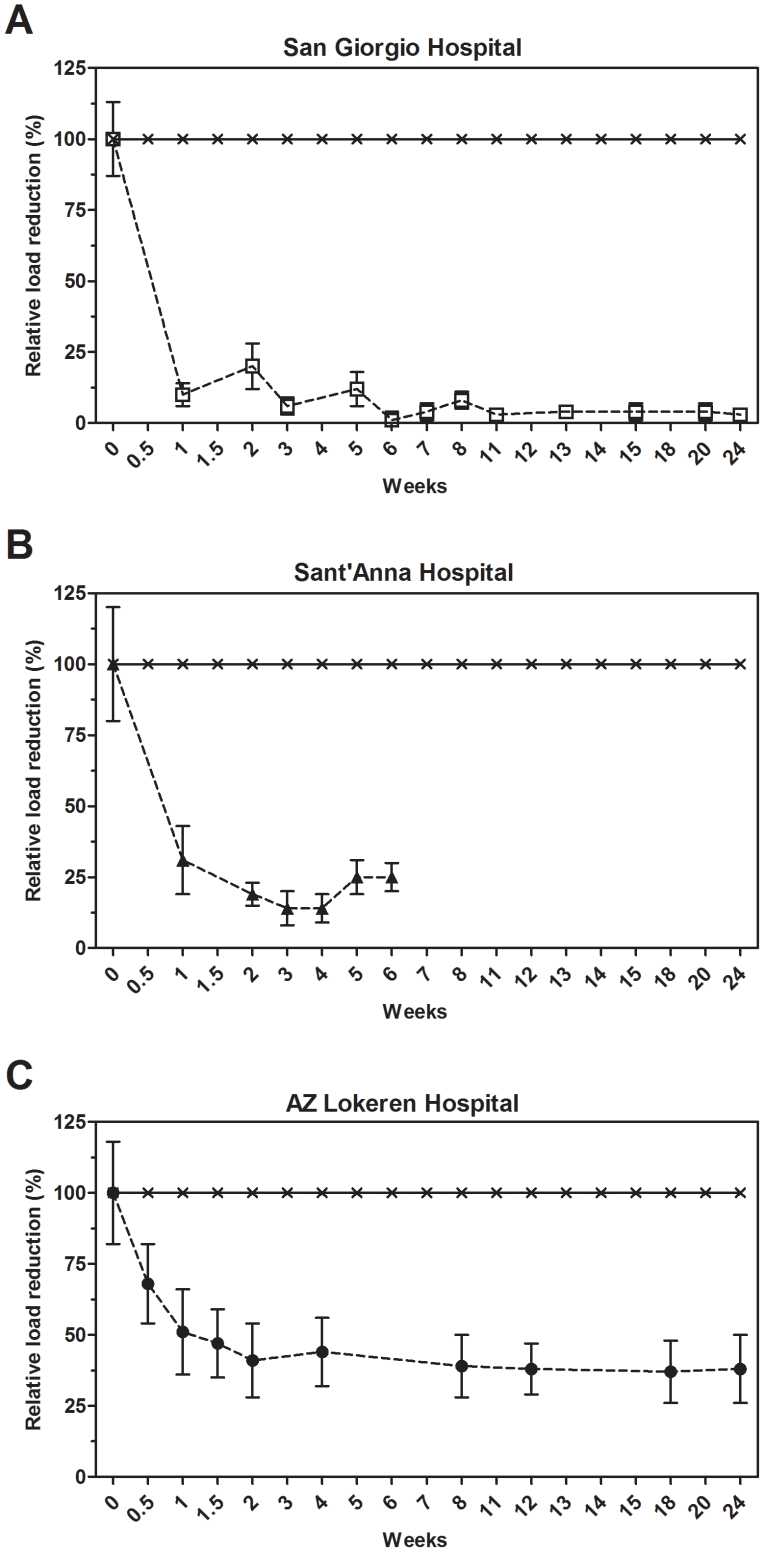
Effect of microbial cleaning on coliforms surface counts. Surface counts for coliforms at the San Giorgio (A), Sant'Anna (B) and AZ Lokeren (C) hospital settings. Results are reported as relative percentage of reduction compared to the control, which was cleaned with conventional (disinfecting) cleaning products. The control is represented by the value of microbial surface contamination (CFU count) at the beginning of the trials (week 0), whose CFU count was set as the 100% in order to obtain reliable comparisons among the three structurally different hospital settings. The analysis indicated that results observed were statistically significant (Table S2).

Figure 1 shows the mean relative values for coliform bacteria load as a general indicator for hygiene. The reference values measured at week 0 for coliforms, with *E.coli* representing about one half of the count, are reported in Table S2. However, individual surface counts could fluctuate strongly, especially in specific areas such as bathrooms or geriatric departments. Noticeably, the beginning of microbial cleaning resulted in a fast reduction of coliform and *E. coli* counts, achieving a maximum effect approximately after two weeks of daily cleaning. The average reduction over time was 74±21% for the coliforms and 89±18% for *E. coli*. The observed reduction was statistically significant for all the monitored hospital settings (Table S2).

### S. aureus

**Figure 2:**
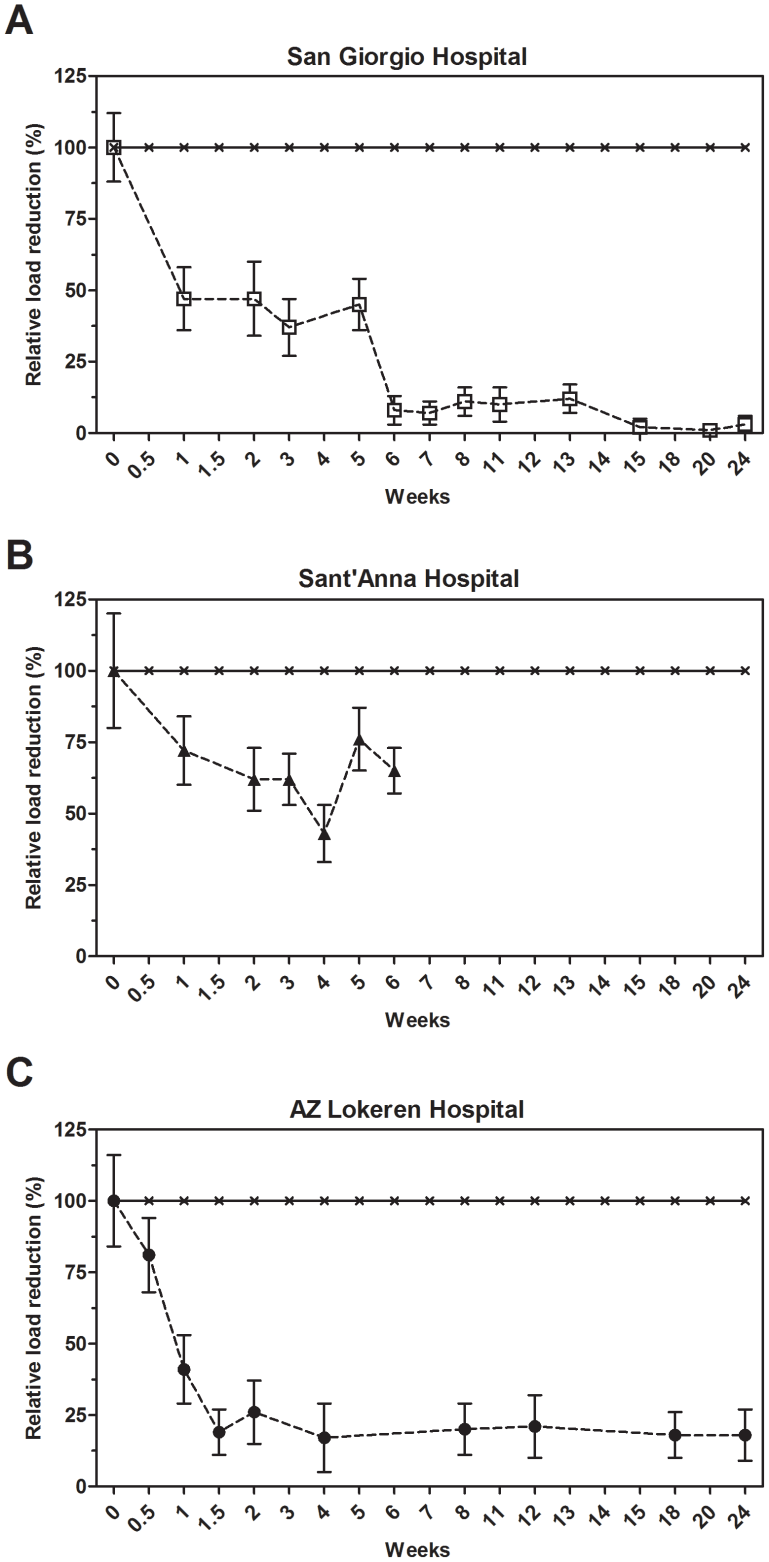
Effect of microbial cleaning on *S. aureus* surface counts. Surface counts for *S. aureus* at the San Giorgio (A), Sant'Anna (B) and AZ Lokeren (C) hospital settings. Results are reported as relative percentage of reduction compared to the control, which was cleaned with conventional (disinfecting) cleaning products. The analysis indicated that results observed were statistically significant (Table S3).

The average levels of *S. aureus* on tested surfaces and the corresponding percentage reduction over time are reported in Table S3 and Figure 2, respectively. For the sake of clarity, no distinction was made between antibiotic-resistant or antibiotic-sensitive *S. aureus*. After 10 days of microbial cleaning within the San Giorgio Hospital setting, the *S. aureus* counts on surfaces dropped, on average, by 58±12%, and 78±15% after six weeks, with a reduction that was highly significant in all the hospital settings (Table S3). In both conventional and microbial cleaning, *S. aureus* counts on treated surfaces showed a good stability with no large exceptions throughout the study (data not shown), suggesting that this organism is not very susceptible to different environmental conditions within a hospital and might therefore survive most common conditions.

### C. difficile

**Figure 3:**
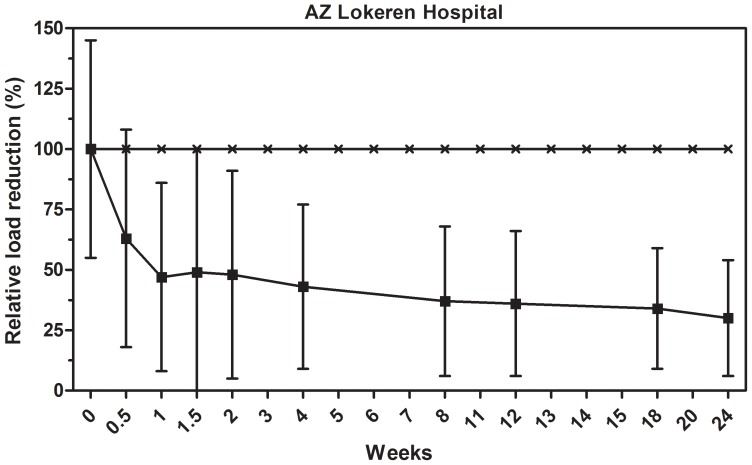
Effect of microbial cleaning on *C. difficile* surface counts. Surface counts are reported as relative percentage of reduction compared to the control, which was cleaned with conventional (disinfecting) cleaning products. The statistical analysis is reported in Table S4.

A less abundant but still common HAI-related organism is *Clostridium difficile*, with average counts of about 500 CFU/m^2^ (Table S4), which is near the detection limit of the test protocols used. These microorganisms were monitored on a regular basis only in the AZ Lokeren setting. *C. difficile* showed much larger specific counts on different surfaces and time points, which, in combination with the overall lower mean counts, resulted in larger standard deviations, thus making the effect of microbial cleaning barely significant when compared to control. The overall average reduction in *C. difficile* load was achieved very quickly after three days of microbial cleaning, with a reduction corresponding to 55±47% (Figure 3). Ongoing measurements in the next 24 weeks indicated that *C. difficile* levels on microbial cleaned surfaces dropped below the detection limit of the analysis method. The observed reduction was significant for weeks 4 (p = 0.048) and 12 (p = 0.007), and particularly for weeks 18 (p<0.0005) and 24 (p = 0.004), indicating the long-term effect on *C. difficile* load reduction.

### C. albicans

**Figure 4:**
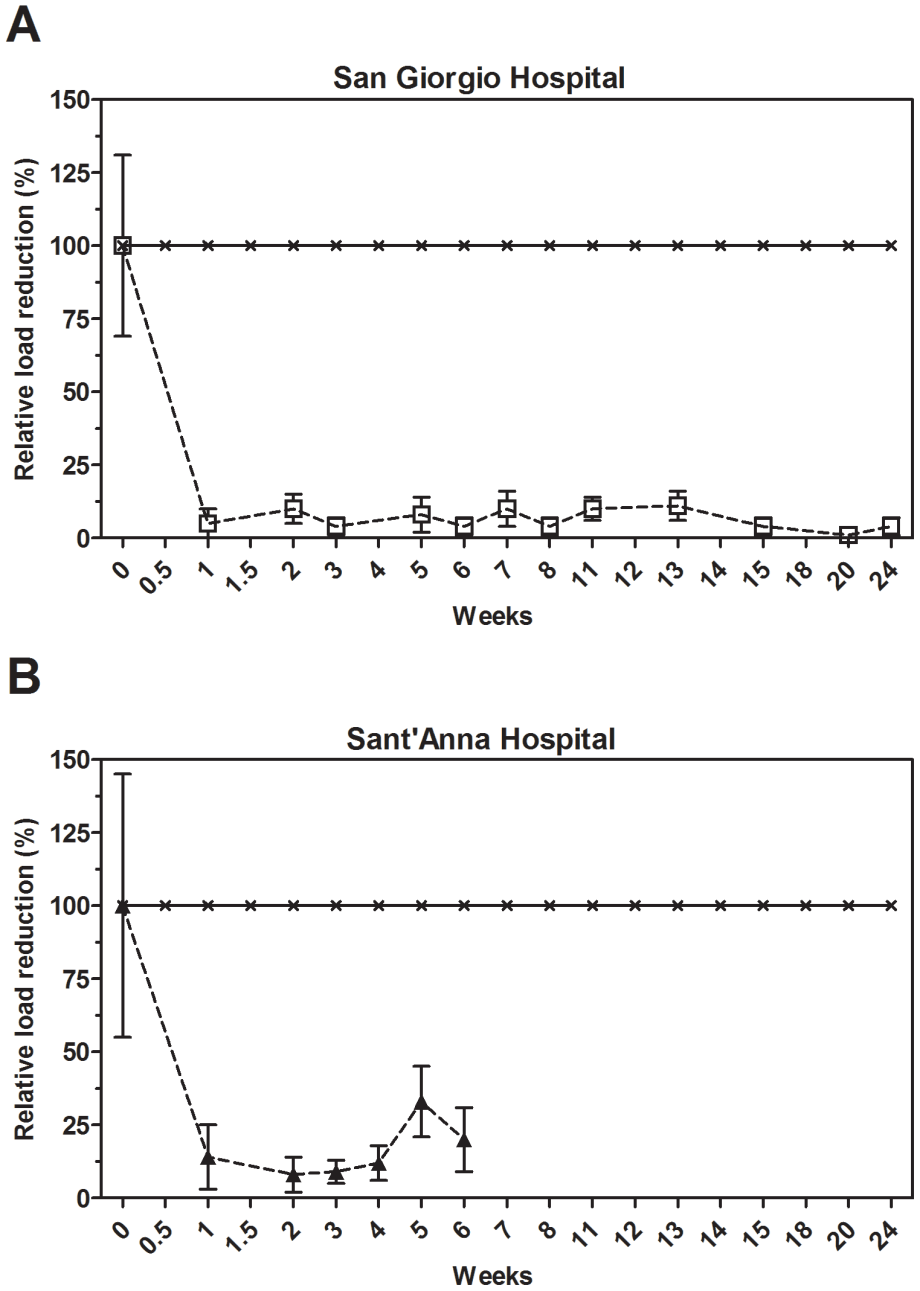
Effect of microbial cleaning on *Candida albicans* surface counts. Surface counts are reported as relative percentage of reduction compared to the control, which was cleaned with conventional (disinfecting) cleaning products. The analysis indicated that results observed were statistically significant (Table S5).

*Candida albicans* load was measured on a regular basis in the two Italian hospital settings. Figure 4 shows the mean relative values of load reduction. The average control values of *C. albicans* are reported in Table S5. Microbial cleaning resulted in a fast load reduction, corresponding to a value of 82±19%, after one week and, noticeably, the load of *C. albicans* was stably maintained at low levels over the next weeks. As of week two, *C. albicans* counts residing on the microbial cleaned surfaces was hardly above the detection limit of the test protocols. Indeed, the observed reduction was highly significant in both the hospital settings (Table S5).

### Time trial with conventional and microbial cleaning

**Figure 5:**
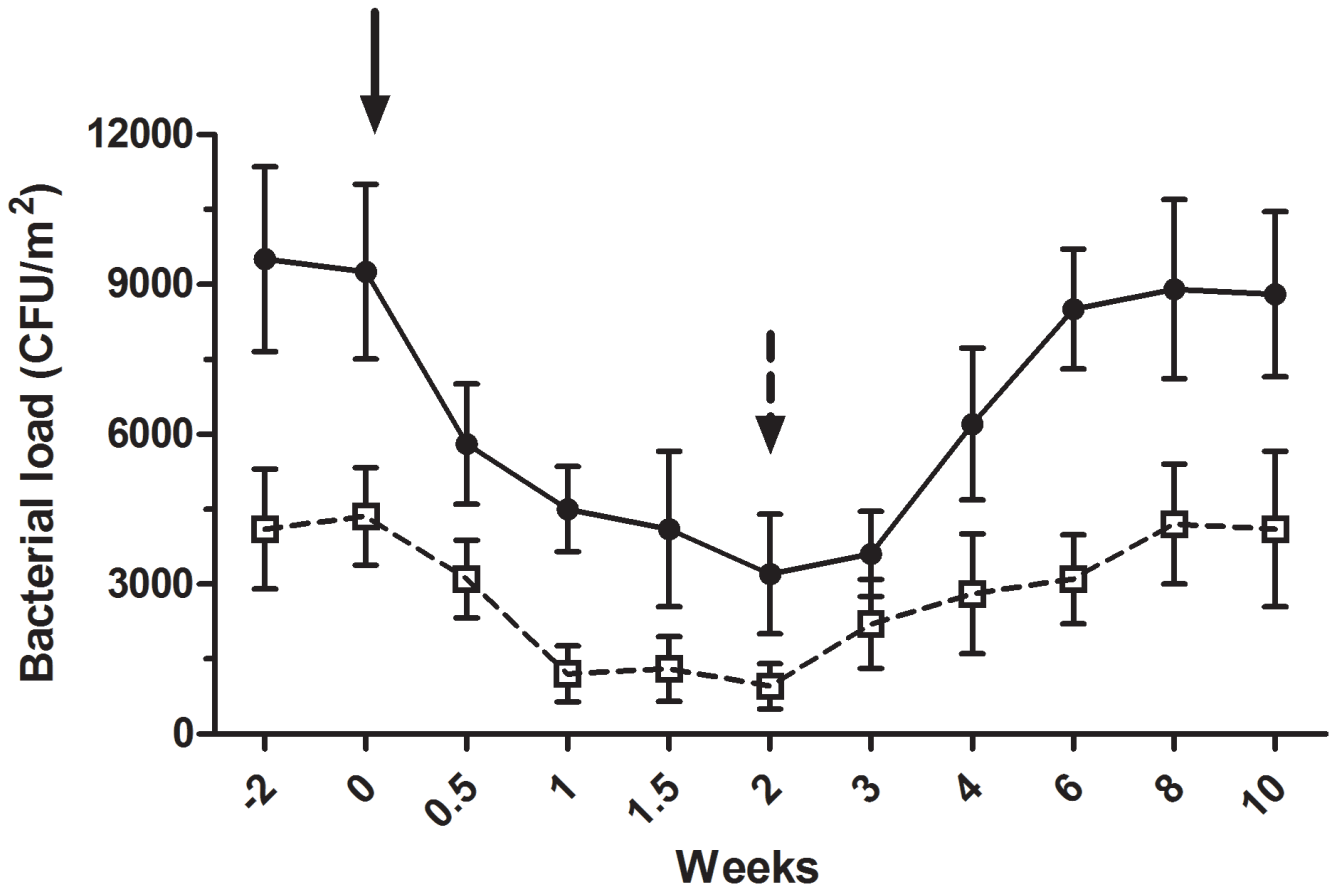
Time course of coliforms and *S. aureus* surface counts. A time-trial of coliforms (black circles) and *S. aureus* (white circles) counts was performed at the geriatrics department of the AZ Lokeren hospital. Surface counts, indicated as CFU/m^2^, were measured after application of conventional (from week −2 to 0) and microbial (from week 0 to 2) cleaning, followed by a subsequent period of conventional cleaning (from week 2 to 10). The application of the probiotic-based products led to a significant decrease in the pathogenic load of coliforms (p<0.0001) and *S. aureus* (p = 0.003). Black arrow: beginning of the microbial cleaning. Black dotted arrow: conventional cleaning.

A separate experiment was conducted within the geriatrics department in the AZ Lokeren setting, where the conventional and microbial cleaning procedures were alternatively applied and the bacterial load was monitored for 10 weeks. Microbial cleaning (from week 0 to week 2) was applied after two weeks of conventional cleaning (from week −2 to week 0), which was then applied for a second period spanning the last 8 weeks (from week 2 to week 10). Consistently with previous observations, the microbial cleaning strongly reduced both coliforms and *S. aureus* load, whose counts dropped from 9250±1750 CFU/m^2^ to 3200±1200 CFU/m^2^ and from 4200±1200 CFU/m^2^ to 950±450 CFU/m^2^ after 2 weeks, respectively. Conversely, when the microbial cleaning was replaced by the conventional procedure, both loads raised to colony counts comparable to those observed before the application of the microbial cleaning (between weeks −2 and 0) (Figure 5). The application of the microbial cleaning led to a significant decrease in the pathogenic load of coliforms (p<0.0001, from week 0.5 to week 3) and *S. aureus* (p = 0.003, from week 1 to week 3) in comparison with that measured at week 0, whereas no significant differences were observed after replacement of the microbial cleaning with the conventional procedure.

Overall, our data on microbial cleaning indicated that the strong reduction of the pathogen load was stably maintained over time, and the observed effect was directly related to its application, as indicated by comparison with conventional cleaning.

### Susceptibility/resistance of Bacillus isolates to antibiotics

In order to monitor the appearance of antimicrobial resistances in *Bacillus* strains, we performed both conventional antibiograms and molecular analyses on *Bacillus* isolates collected on the treated surfaces. Preliminary results from antibiograms showed that the Bacilli tested, deriving from both the PCHS products and from isolates, displayed the known natural resistance to penicillin. The susceptibility profile for the other antibiotics tested (cefoperazone, cefalotin and gentamicin) was comparable to that of the ATCC reference strain, with the only exception of clindamycin, for which intermediate values were observed (Table 1).

**Table 1:** Antibiograms on *Bacillus spp.* from the cleaning products and isolates.

	Zone Diameter Interpretive Criteria (mm)				
	Beta-lactams			Aminoglycosides	Lincosamides
	Penicillin	Cefoperazone	Cefalotin	Gentamicin	Clindamycin
	(10 U)	(30 µg)	(30 µg)	(10 µg)	(2 µg)
ATCC 6633	7 R^a^	20 I^b^	30 S	25 S	25 S
PCHS product	14 R	19 I	24 S	24 S	18 I
*Bacillus spp.* isolate 1	12 R	19 I	20 S	25 S	20 I
*Bacillus spp.* isolate 2	15 R	20 I	30 S	25 S	16 I
*Bacillus spp.* isolate 3	10 R	30 S^c^	35 S	30 S	25 S

a Resistant.

b Intermediate.

c Susceptible.

*Note*: for reference criteria see Table S1.

Antibiograms were conducted on *Bacilli* from the PCHS products and isolates, with ATCC 6633 strain as reference. Results are reported as diameter (expressed in mm) of the zones of inhibition and the corresponding interpretive criteria, in accordance to the CLSI reference criteria [38] (Table S1).

To increase the number of antimicrobial resistance factors analyzed, 20 *Bacillus* isolates, which were collected from 6 to 12 months after the beginning of the hospital trial, were also analyzed by a qPCR assay capable of detecting 87 different genes associated to antibiotic resistance. Prior to testing *Bacillus* isolates, the method was set up on known *Bacillus spp.* DNA samples, which indicated this technique as a reliable tool for subsequent analysis.

**Figure 6:**
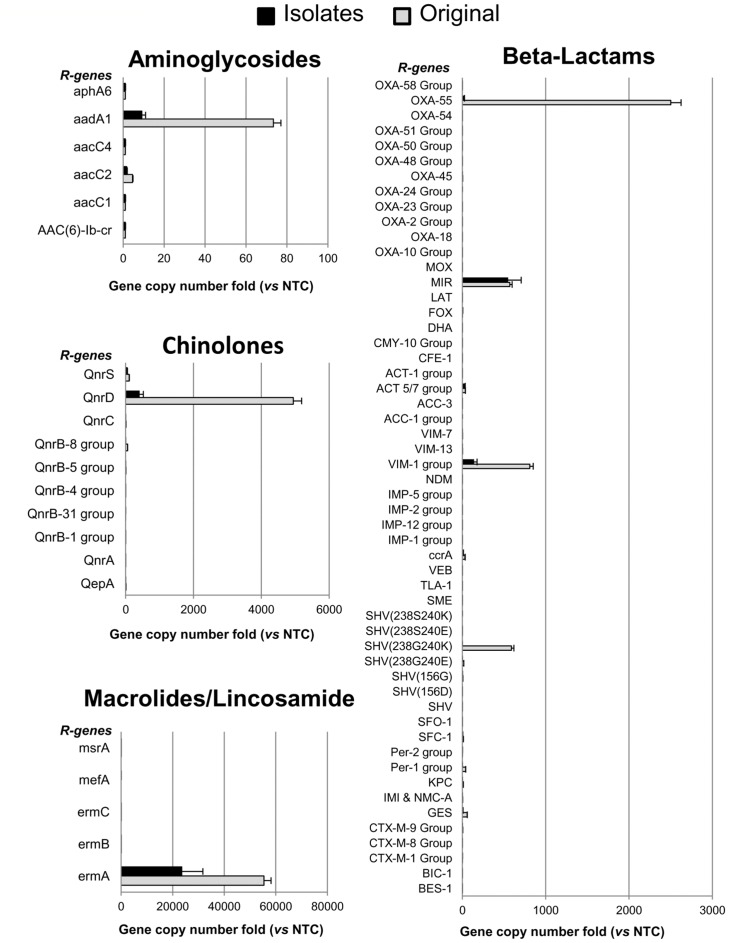
Antimicrobial resistance genes profile by qPCR assay. The DNA of the *Bacillus spp.* from the cleaning products (Original) and from 20 *Bacillus spp.* colonies isolated from the treated surfaces up to 12 months after the beginning of the cleaning protocol (Isolates), was analyzed by qPCR Microarray to detect the presence of antibiotic resistance genes (R genes). DNA from *E. coli* JM101 strain was used as negative control (NTC). Results are reported as folds of gene copy number comparing the detected values with those obtained in the negative control DNA, both normalized for bacterial cell number. Those genes coding for antibiotics that are not included in the figure (i.e. vancomycin, tetracyclins) were negative both in Original and Isolates collections. Results are reported as mean ± standard deviation.

The analysis of the probiotic-based cleaning products revealed the existence of constitutive resistance genes in the *Bacillus* species contained in the original product formulation, confirming previous literature reports (Figure 6).

Noticeably, new or acquired resistance genes were completely absent in all the *Bacillus* isolates tested, indicating that these bacteria did not undergo mutagenicity or gene transfer events, even after 12 months after application, thus confirming the results observed in the antibiogram assays.

## Discussion

Given the latest official reports on the prevalence of HAIs [3] and the evolution of microbial resistance to antibiotics [39] and disinfectants [20], it is clear that new and sustainable strategies to address contaminated surfaces in hospital settings are of great interest. Besides measures that deal with person-to-person transmission of pathogens, contaminated environmental surfaces provide an important potential source for transmission of many healthcare-associated pathogens due to their persistence on such inanimate surfaces [9], [10], [40]. This highlights the importance of cleaning procedures aimed to control the load of pathogenic bacteria that reside on hospital surfaces and, as a consequence, of HAIs, as indicated by considerable evidence about the benefits of hospital cleanliness towards reducing HAIs [18]

This study exploited the hypothesis proposed by Falagas & Makris [22] to use non-pathogenic microorganisms, namely probiotics, as part of daily cleaning products to lower the incidence of HAI-related microorganisms in healthcare facilities. Evidences on the efficacy of probiotics for the prevention and treatment of infections have been observed both in vitro and in vivo [41]–[43].

A variety of HAI-related microorganisms exist and for this study the selection was mostly determined by the relevance and relative abundance of the microorganisms in the hospitals willing to participate to the present study. Coliforms were selected as an indicator for overall hygiene and cleaning assessment. Although some HAIs are associated with outbreaks and others show rather constant prevalence, the related microorganisms can be found on hard surfaces at any time as presented in this study. This implies that surfaces act as a reservoir that might initiate an outbreak when people get contaminated and start spreading the pathogens amongst each other.

The aim of this study was to collect a substantial amount of data from different independent healthcare facilities and the 20000 microbial surface samples contribute to the significance of the results. However, the amount of analyses makes it impossible to present all specific data and observations in this study. Therefore, given the rather compatible mean microbial counts between the several hospitals, the witnessed trends in surface microbiology upon microbial cleaning was presented.

In general, it was observed that microbial cleaning of hard surfaces resulted in a clear change of the microbiology within two weeks. With the exception of *Clostridium difficile*, a very significant decrease in HAI-related microorganisms, compared to conventional cleaning and disinfection, was observed. As of two weeks of microbial cleaning, the reduced HAI-related microorganisms count remained constant and mostly just above detection limit of the test protocol. The standard deviation of some values was high (likely due to large differences in the type of tested surfaces), but still the difference was clearly significant to demonstrate the effects exerted by the microbial cleaning compared to the control. Our results indicate that microbial cleaning clearly lowered the presence of HAI-associated microorganisms on hard surfaces compared to conventional cleaning procedures, without significantly lowering the total microbial counts. In addition, our data show that when microbial cleaning was switched to the conventional procedure, the HAI-related microorganism counts raised to the original (higher) levels observed before the beginning of microbial cleaning. This observation indicates that microbial cleaning has a time-limited effect on the counts of HAI-related microorganisms and that microbial cleaning needs to be continuously applied on regular basis in order to stably maintain the reduced levels of these microorganisms. Microbial cleaning therefore resulted in the partial non-permanent replacement of HAI-related microorganisms by the *Bacillus* strains used in the microbial cleaning products.

Although this study did not aim to investigate the actual mechanisms involved in the observed changes in surface microbiota, some assumptions can be made based on existing literature. The most relevant mechanism underlying the observed effect is likely due to competitive exclusion: *Bacillus* strains applied on the surfaces within the cleaning solution may compete, in terms of nutrients and space, with the microbiota already present on the surfaces [44]. Woo & Ahn have suggested that competitive exclusion can also destabilize certain biofilms [44], which was observed in the present study as removal of grout on certain hard surfaces after several weeks of microbial cleaning. Such biofilm removal on hard surfaces by means of microbial cleaning deserves further research. Another mechanism that is likely to contribute to the changes in microbiota upon microbial cleaning, and also destabilize biofilm formation, is quorum sensing and quorum quenching [45]. The constant artificial introduction of dominant counts of *Bacillus spp.* through the cleaning products could destabilize the microbial population dynamics on surfaces and in biofilm (e.g. grout). Given the observed general effect of microbial cleaning on several HAI-related microorganisms, it is likely that non-selective effects such as competitive exclusion and quorum sensing are the most important mechanisms involved. Besides these general mechanisms, other more specific interactions could contribute to the reduction effect on one or more HAI-related microorganisms. For instance, the production of either bacteriocins [46] or some enzymes known to be produced by *Bacillus spp.* might affect other organisms to a higher extent. Regarding *Clostridium*, the authors suggest that the cortex-lytic enzymes involved in the germination of the *Bacillus* spores [47] in the microbial cleaning products might also trigger the germination of *Clostridium* spores on surfaces. The germinated *Clostridium* cells might subsequently be killed by oxygen or could suffer from competitive exclusion. Regardless the actual mechanisms involved, this study demonstrates that microbial cleaning in healthcare facilities manages to significantly lower the surface counts of a variety of HAI-associated microorganisms.

The most important question concerns the safety of applying microbial cleaning as biocontrol system in healthcare facilities. Obviously the identification and safety assessment of the bacterial strains used in the products is of utmost importance, as well as the production processes involved throughout the whole production chain. The strains used in this study (*Bacillus subtilis*, *Bacillus pumilus* and *Bacillus megaterium*), were food grade organisms, for which substantial safety and toxicity data existed by the manufacturer, and are also known to be not harmful to humans [48]–[51]. In addition, all production and quality control processes were ISO9001:2008 certified. Furthermore, the use of probiotics for several biotechnological and biopharmaceutical applications, as indicated by the very recent employment of *B. subtilis* as a biocontrol agent in aquaculture [52], agriculture [53]–[55], as adjuvant [56], [57] or, in the spore form, as delivery system for development of new vaccines [58]–[63] has been subject to study and control.

However, it is clear that microbial cleaning alone will not solve the emerging HAI problem. There is strong evidence indicating that a correct hand hygiene program in healthcare settings represents an effective behavior for the purpose of reducing HAIs [64], [65]. Therefore, microbial cleaning needs to be part of a global hygiene protocol, such as the proposed microbial cleaning system (PCHS) used in this study. Such protocol outlines in which specific areas or events a proper disinfection is required over microbial cleaning, as well as the instructions to proper hand hygiene or isolation of contaminated persons. Also, sporadic outbreaks such as viral infections might ask for exceptional measures that do not follow the routine cleaning or disinfection procedures. Despite the reductive effect on several HAI-related microorganisms, microbial cleaning products are not to be used or considered as disinfectants. Indeed, when a surface is actively contaminated, disinfection is needed, in particular for those surface that are located in high risk areas. Cleaning with probiotic-based products can overcome the limited action of traditional disinfectants by decreasing the re-occurrence of pathogenic loads on surfaces and by removing biofilms that can act as a shelter for other pathogens. The probiotic-based products used in this study are suitable only for cleaning, thus indicating that when a disinfection is really necessary, a disinfectant must be used, but the combination with probiotic-based cleaning will allow optimal long-term maintenance of low levels of contamination.

In order to evaluate the susceptibility or resistance of the *Bacillus* strains to antibiotics, we recently implemented our research by exploiting antibiogram tests, which have been performed on colonies of *Bacillus spp.* coming from the same sample surfaces (e.g. floor) monitored in the hospital settings. Our first results indicate that the isolated *Bacillus spp.* strains are susceptible to the antibiotics tested, with the exception of those towards which the *Bacillus spp.* is naturally resistant, as also indicated by antibiograms performed on the standard ATCC strain.

We are aware that the study was focused on a limited number of microorganisms, and that culture-dependent techniques display some limitations, due to the fact that single colonies might be not fully representative of the bacterial genus analyzed. As to safety concerns, in order to increase the representativeness of the results, 20 *Bacillus* isolates were also tested by a qPCR assay, which was designed to simultaneously assess the presence of 87 different resistance genes. Beside the high number of resistance factors analyzed, this method takes advantage of a high sensitivity, which permits the detection of very few copies of the target gene, thus allowing the identification of antibiotic resistance genes even when only a very low number of bacterial cells within the sample are positive to antibiotic resistance. The reliability of this assay for the analysis of *Bacillus* strains is supported by the detection, as expected, of those resistance genes known to be present in the *Bacillus spp.* contained in the formulation of the cleaning product used [66]. Although these assays were performed on only 20 *Bacillus spp*. isolates, these preliminary results show that they did not display acquisition of new resistance genes, even in a period of 12 months, thus strengthening the hypothesis that the use of *Bacillus* spores in cleaning products might be considered as safe. On the other hand, results also show that this method can be reliable for the evaluation of antimicrobial resistance factors in *Bacillus*, and designate this assay as an important implementation for future studies also focused on safety concerns.

## Conclusions

Given the recent and fast evolution of multi-resistant pathogens in healthcare facilities there is a need for sustainable and effective alternatives to the cleaning and disinfection chemicals used today. This study demonstrates that a microbial (probiotic-based) cleaning is more effective in the long-term lowering of the number of HAI-related microorganisms on surfaces, when compared to conventional cleaning products, even those containing disinfectant molecules such as chlorine. The first indications on the percentages of HAIs in the trial hospitals monitored on a continuous basis throughout the study are very promising and may pave the way for a novel and cost-effective strategy to counteract or (bio)control healthcare-associated pathogens.

When it comes down to risk management, one has to decide whether a patient should stay in an environment dominated by food grade microorganisms or in an environment harboring an elevated level of increasingly resistant pathogens.

## Supporting Information

Table S1 **Reference criteria for interpretation of the zones of inhibition in the disk diffusion antimicrobial susceptibility test.** The table shows the disk diffusion interpretive criteria for the correlation of the diameter (expressed in mm) of the zones of inhibition with the corresponding interpretation, referred as Susceptible (S), Intermediate (I) and Resistant (R), according to the CLSI reference criteria [38].
(XLSX)

Table S2 **Statistical analysis on the reduction of coliforms load.** Results are expressed as CFU/m^2^.
(XLSX)

Table S3 **Statistical analysis on the reduction of** ***Staphylococcus aureus*** **load.** Results are expressed as CFU/m^2^.
(XLSX)

Table S4 **Statistical analysis on the reduction of** ***Clostridium difficile*** **load.** Results are expressed as CFU/m^2^.
(XLSX)

Table S5 **Statistical analysis on the reduction of** ***Candida albicans*** **load.** Results are expressed as CFU/m^2^.
(XLSX)

## References

[1] Burke JP (2003) Infection control - a problem for patient safety. N Engl J Med 348: 651–656. 12584377 10.1056/NEJMhpr020557

[2] Allegranzi B, Nejad SB, Castillejos GG, Kilpatrick C, Kelley E, et al. Clean Care is Safer Care Team (2011) Report on the Burden of Endemic Health Care–Associated Infection Worldwide. Geneva, Switzerland: World Health Organization. Available: http://whqlibdoc.who.int/publications/2011/9789241501507_eng.pdf

[3] Suetens C, Hopkins S, Kolman J, Diaz Högberg L (2013) Point prevalence survey of healthcare associated infections and antimicrobial use in European acute care hospitals. Stockholm, Sweden: European Centre for Disease Prevention and Control. Available: http://www.ecdc.europa.eu/en/publications/publications/healthcare-associated-infections-antimicrobial-use-pps.pdf.

[4] Klevens RM, Edwards JR, Richards CL Jr, Horan TC, Gaynes RP, et al (2007) Estimating health care-associated infections and deaths in U.S. hospitals, 2002. Public Health Rep 122: 160–166. 17357358 10.1177/003335490712200205 PMC1820440

[5] Scott D (2009) The direct medical costs of Healthcare-Associated Infections in U.S. Hospitals and the benefits of prevention. Atlanta: Centers for Disease Control and Prevention (CDC). Available: http://www.cdc.gov/hai/pdfs/hai/scott_costpaper.pdf.

[6] Davis GS, Sevdalis N, Drumright LN (2014) Spatial and temporal analyses to investigate infectious disease transmission within healthcare settings. J Hosp Infect 86: 227–243. 24650720 10.1016/j.jhin.2014.01.010 PMC7133762

[7] Gaudart J, Cloutman-Green E, Guillas S, D'Arcy N, Hartley JC, et al (2013) Healthcare environments and spatial variability of healthcare associated infection risk: cross-sectional surveys. PLoS One 8: e76249. 24069459 10.1371/journal.pone.0076249 PMC3777895

[8] Hota B (2004) Contamination, disinfection, and cross-colonization: are hospital surfaces reservoirs for nosocomial infection? Clin Infect Dis 39: 1182–1189. 15486843 10.1086/424667 PMC7107941

[9] Kramer A, Schwebke I, Kampf G (2006) How long do nosocomial pathogens persist on inanimate surfaces? A systematic review. BMC Infect Dis 6: 130. 16914034 10.1186/1471-2334-6-130 PMC1564025

[10] Otter JA, Yezli S, Salkeld JA, French GL (2013) Evidence that contaminated surfaces contribute to the transmission of hospital pathogens and an overview of strategies to address contaminated surfaces in hospital settings. Am J Infect Control 41: S6–11. 23622751 10.1016/j.ajic.2012.12.004

[11] Rutala WA, Weber DJ (2005) The benefits of surface disinfection. Am J Infect Control 33: 434–435. 16153494 10.1016/j.ajic.2004.12.007

[12] Dettenkofer M, Spencer RC (2007) Importance of environmental decontamination–a critical view. J Hosp Infect 65 Suppl 2: 55–57. 17540243 10.1016/S0195-6701(07)60016-4

[13] Rutala WA (1996) APIC guideline for selection and use of disinfectants. 1994, 1995, and 1996 APIC Guidelines Committee. Association for Professionals in Infection Control and Epidemiology, Inc. Am J Infect Control 24: 313–342. 8870916 10.1016/s0196-6553(96)90066-8

[14] Mangram AJ, Horan TC, Pearson ML, Silver LC, Jarvis WR (1999) Guideline for prevention of surgical site infection, 1999. Hospital Infection Control Practices Advisory Committee. Infect Control Hosp Epidemiol 20: 250–278. 10219875 10.1086/501620

[15] WHO/CDS/CSR/EPH. Prevention of Hospital-acquired infections; a practical guide. Vol. 12, 2002.

[16] Sehulster L, Chinn RY (2003) Cdc, Hicpac (2003) Guidelines for environmental infection control in health-care facilities. Recommendations of CDC and the Healthcare Infection Control Practices Advisory Committee (HICPAC). MMWR Recomm Rep 52: 1–42. 12836624

[17] Rutala WA, Weber DJ, and the Healthcare Infection Control Practices Advisory Committee (HICPAC) (2008) Guidelines for disinfection and sterilization in healthcare facilities. http://www.cdc.gov/hicpac/Disinfection_Sterilization/toc.html, Centers for Disease Control and Prevention (CDC).

[18] Dancer SJ (2009) The role of environmental cleaning in the control of hospital-acquired infection. J Hosp Infect 73: 378–385. 19726106 10.1016/j.jhin.2009.03.030

[19] Weber DJ, Rutala WA (2013) Assessing the risk of disease transmission to patients when there is a failure to follow recommended disinfection and sterilization guidelines. Am J Infect Control 41: S67–71. 23622753 10.1016/j.ajic.2012.10.031

[20] McDonnell G, Russell AD (1999) Antiseptics and disinfectants: activity, action, and resistance. Clin Microbiol Rev 12: 147–179. 9880479 10.1128/cmr.12.1.147 PMC88911

[21] Frabetti A, Vandini A, Balboni P, Triolo F, Mazzacane S (2009) Experimental evaluation of the efficacy of sanitation procedures in operating rooms. Am J Infect Control 37: 658–664. 19595481 10.1016/j.ajic.2009.03.011

[22] Falagas ME, Makris GC (2009) Probiotic bacteria and biosurfactants for nosocomial infection control: a hypothesis. J Hosp Infect 71: 301–306. 19201053 10.1016/j.jhin.2008.12.008

[23] Gause GF (1932) Experimental studies on the struggle for existence. J Exp Biol 9: 389–402.

[24] Hardin G (1960) The competitive exclusion principle. Science 131: 1292–1297. 14399717 10.1126/science.131.3409.1292

[25] Hibbing ME, Fuqua C, Parsek MR, Peterson SB (2010) Bacterial competition: surviving and thriving in the microbial jungle. Nat Rev Microbiol 8: 15–25. 19946288 10.1038/nrmicro2259 PMC2879262

[26] Gatesoupe FJ (1999) The use of probiotics in aquaculture. Aquaculture 180: 147–165.

[27] Temmerman R, Vervaeren H, Noseda B, Boon N, Verstraete W (2007) Inhibition of *Legionella pneumophila* by *Bacillus sp* . Eng Life Science 7: 1–8.

[28] Rodrigues L, van der Mei H, Teixeira JA, Oliveira R (2004) Biosurfactant from *Lactococcus lactis* 53 inhibits microbial adhesion on silicone rubber. Appl Microbiol Biotechnol 66: 306–311. 15290139 10.1007/s00253-004-1674-7

[29] Rodrigues L, van der Mei HC, Teixeira J, Oliveira R (2004) Influence of biosurfactants from probiotic bacteria on formation of biofilms on voice prostheses. Appl Environ Microbiol 70: 4408–4410. 15240331 10.1128/AEM.70.7.4408-4410.2004 PMC444786

[30] Rodrigues L, van der Mei H, Banat IM, Teixeira J, Oliveira R (2006) Inhibition of microbial adhesion to silicone rubber treated with biosurfactant from *Streptococcus thermophilus A* . FEMS Immunol Med Microbiol 46: 107–112. 16420603 10.1111/j.1574-695X.2005.00006.x

[31] Rodrigues LR, Banat IM, van der Mei HC, Teixeira JA, Oliveira R (2006) Interference in adhesion of bacteria and yeasts isolated from explanted voice prostheses to silicone rubber by rhamnolipid biosurfactants. J Appl Microbiol 100: 470–480. 16478486 10.1111/j.1365-2672.2005.02826.x

[32] Walencka E, Rozalska S, Sadowska B, Rozalska B (2008) The influence of *Lactobacillus acidophilus*-derived surfactants on staphylococcal adhesion and biofilm formation. Folia Microbiol (Praha) 53: 61–66. 18481220 10.1007/s12223-008-0009-y

[33] Bauer AW, Perry DM, Kirby WM (1959) Single-disk antibiotic-sensitivity testing of staphylococci; an analysis of technique and results. A M A Arch Intern Med 104: 208–216. 13669774 10.1001/archinte.1959.00270080034004

[34] Kirby WM, Yoshihara GM, Sundsted KS, Warren JH (1957) Clinical usefulness of a single disc method for antibiotic sensitivity testing. Antibiot Annu 1956–1957: 892–897. 13425478

[35] Bauer AW, Kirby WM, Sherris JC, Turck M (1966) Antibiotic susceptibility testing by a standardized single disk method. Am J Clin Pathol 45: 493–496. 5325707

[36] CLSI M45A (2006) Methods for antimicrobial dilution and disk susceptibility testing of infrequently isolated or fastidious bacteria. Approved Guideline. Wayne, PA: Clinical Laboratory Standard Institute (CLSI).

[37] CLSI M2-A9 (2009) Performance standards for antimicrobial disk susceptibility tests; Approved standard. 9th ed. Wayne, PA: Clinical Laboratory Standards Institute (CLSI).

[38] CLSI M100-S20 (2010) Performance Standards for Antimicrobial Susceptibility Testing; 20th Informational Supplement. Wayne, PA: Clinical Laboratory Standards Institute (CLSI).

[39] Cohen NR, Lobritz MA, Collins JJ (2013) Microbial persistence and the road to drug resistance. Cell Host Microbe 13: 632–642. 23768488 10.1016/j.chom.2013.05.009 PMC3695397

[40] Donskey C (2013) Does improving surface cleaning and disinfection reduce health care-associated infections? Am J Infect Control 41: S12–S19. 23465603 10.1016/j.ajic.2012.12.010

[41] Shu M, Wang Y, Yu J, Kuo S, Coda A, et al (2013) Fermentation of *Propionibacterium acnes*, a commensal bacterium in the human skin microbiome, as skin probiotics against methicillin-resistant *Staphylococcus aureus* . PLoS One 8: e55380. 23405142 10.1371/journal.pone.0055380 PMC3566139

[42] Levkovich T, Poutahidis T, Smillie C, Varian BJ, Ibrahim YM, et al (2013) Probiotic bacteria induce a ‘glow of health’. PLoS One 8: e53867. 23342023 10.1371/journal.pone.0053867 PMC3547054

[43] Ohlsson C, Engdahl C, Fak F, Andersson A, Windahl SH, et al (2014) Probiotics protect mice from ovariectomy-induced cortical bone loss. PLoS One 9: e92368. 24637895 10.1371/journal.pone.0092368 PMC3956931

[44] Woo J, Ahn J (2013) Probiotic-mediated competition, exclusion and displacement in biofilm formation by food-borne pathogens. Lett Appl Microbiol 56: 307–313. 23362863 10.1111/lam.12051

[45] Christiaen SE, Matthijs N, Zhang XH, Nelis HJ, Bossier P, et al (2014) Bacteria that inhibit quorum sensing decrease biofilm formation and virulence in *Pseudomonas aeruginosa* PAO1. Pathog Dis 70: 271–279. 24415453 10.1111/2049-632X.12124

[46] Beric T, Stankovic S, Draganic V, Kojic M, Lozo J, et al (2013) Novel antilisterial bacteriocin licheniocin 50.2 from *Bacillus licheniformis* VPS50.2 isolated from soil sample. J Appl Microbiol 10.1111/jam.12393 24238327

[47] Ustok FI, Packman LC, Lowe CR, Christie G (2014) Spore germination mediated by *Bacillus megaterium* QM B1551 SleL and YpeB. J Bacteriol 196: 1045–1054. 24375103 10.1128/JB.01298-13 PMC3957701

[48] de Boer AS, Diderichsen B (1991) On the safety of *Bacillus subtilis* and *B. amyloliquefaciens*: a review. Appl Microbiol Biotechnol 36: 1–4. 1367772 10.1007/BF00164689

[49] Leonel Ochoa-Solano J, Olmos-Soto J (2006) The functional property of *Bacillus* for shrimp feeds. Food Microbiol 23: 519–525. 16943046 10.1016/j.fm.2005.10.004

[50] Vary PS, Biedendieck R, Fuerch T, Meinhardt F, Rohde M, et al (2007) *Bacillus megaterium*–from simple soil bacterium to industrial protein production host. Appl Microbiol Biotechnol 76: 957–967. 17657486 10.1007/s00253-007-1089-3

[51] Porwal S, Lal S, Cheema S, Kalia VC (2009) Phylogeny in aid of the present and novel microbial lineages: diversity in *Bacillus* . PLoS One 4: e4438. 19212464 10.1371/journal.pone.0004438 PMC2639701

[52] Ran C, Carrias A, Williams MA, Capps N, Dan BC, et al (2012) Identification of *Bacillus* strains for biological control of catfish pathogens. PLoS One 7: e45793. 23029244 10.1371/journal.pone.0045793 PMC3448714

[53] Al-Ajlani MM, Sheikh MA, Ahmad Z, Hasnain S (2007) Production of surfactin from *Bacillus subtilis* MZ-7 grown on pharmamedia commercial medium. Microb Cell Fact 6: 17. 17550616 10.1186/1475-2859-6-17 PMC1894814

[54] Zhao Y, Selvaraj JN, Xing F, Zhou L, Wang Y, et al (2014) Antagonistic Action of *Bacillus subtilis* Strain SG6 on *Fusarium graminearum* . PLoS One 9: e92486. 24651513 10.1371/journal.pone.0092486 PMC3961383

[55] Adam M, Heuer H, Hallmann J (2014) Bacterial antagonists of fungal pathogens also control root-knot nematodes by induced systemic resistance of tomato plants. PLoS One 9: e90402. 24587352 10.1371/journal.pone.0090402 PMC3938715

[56] Barnes AG, Cerovic V, Hobson PS, Klavinskis LS (2007) *Bacillus subtilis* spores: a novel microparticle adjuvant which can instruct a balanced Th1 and Th2 immune response to specific antigen. Eur J Immunol 37: 1538–1547. 17474150 10.1002/eji.200636875

[57] de Souza RD, Batista MT, Luiz WB, Cavalcante RC, Amorim JH, et al (2014) *Bacillus subtilis* spores as vaccine adjuvants: further insights into the mechanisms of action. PLoS One 9: e87454. 24475289 10.1371/journal.pone.0087454 PMC3903701

[58] Huang JM, Hong HA, Van Tong H, Hoang TH, Brisson A, et al (2010) Mucosal delivery of antigens using adsorption to bacterial spores. Vaccine 28: 1021–1030. 19914191 10.1016/j.vaccine.2009.10.127

[59] Lee S, Belitsky BR, Brown DW, Brinker JP, Kerstein KO, et al (2010) Efficacy, heat stability and safety of intranasally administered *Bacillus subtilis* spore or vegetative cell vaccines expressing tetanus toxin fragment C. Vaccine 28: 6658–6665. 20709005 10.1016/j.vaccine.2010.08.016

[60] Amuguni JH, Lee S, Kerstein KO, Brown DW, Belitsky BR, et al (2011) Sublingually administered *Bacillus subtilis* cells expressing tetanus toxin C fragment induce protective systemic and mucosal antibodies against tetanus toxin in mice. Vaccine 29: 4778–4784. 21565244 10.1016/j.vaccine.2011.04.083

[61] Permpoonpattana P, Hong HA, Phetcharaburanin J, Huang JM, Cook J, et al (2011) Immunization with Bacillus spores expressing toxin A peptide repeats protects against infection with Clostridium difficile strains producing toxins A and B. Infect Immun 79: 2295–2302. 21482682 10.1128/IAI.00130-11 PMC3125831

[62] Amuguni H, Lee S, Kerstein K, Brown D, Belitsky B, et al (2012) Sublingual immunization with an engineered *Bacillus subtilis* strain expressing tetanus toxin fragment C induces systemic and mucosal immune responses in piglets. Microbes Infect 14: 447–456. 22198093 10.1016/j.micinf.2011.12.001

[63] Hinc K, Stasilojc M, Piatek I, Peszynska-Sularz G, Isticato R, et al (2014) Mucosal Adjuvant Activity of IL-2 Presenting Spores of Bacillus subtilis in a Murine Model of Helicobacter pylori Vaccination. PLoS One 9: e95187. 24743850 10.1371/journal.pone.0095187 PMC3990602

[64] Chen YC, Sheng WH, Wang JT, Chang SC, Lin HC, et al (2011) Effectiveness and limitations of hand hygiene promotion on decreasing healthcare-associated infections. PLoS One 6: e27163. 22110610 10.1371/journal.pone.0027163 PMC3217962

[65] Pittet D, Hugonnet S, Harbarth S, Mourouga P, Sauvan V, et al (2000) Effectiveness of a hospital-wide programme to improve compliance with hand hygiene. Infection Control Programme. Lancet 356: 1307–1312. 11073019 10.1016/s0140-6736(00)02814-2

[66] Larsen N, Stuer-Lauridsen B, Cantor MD, Nielsen B, Brockmann E, et al (2014) Characterization of Bacillus spp. strains for use as a probiotic additives in pig feed. Appl Microbiol Biotechnol 98: 1105–1118. 24201893 10.1007/s00253-013-5343-6

